# Using implementation mapping to develop strategies for preventing non-communicable diseases in Japanese small- and medium-sized enterprises

**DOI:** 10.3389/fpubh.2022.873769

**Published:** 2022-10-06

**Authors:** Miyuki Odawara, Junko Saito, Akiko Yaguchi-Saito, Maiko Fujimori, Yosuke Uchitomi, Taichi Shimazu

**Affiliations:** ^1^Division of Behavioral Sciences, National Cancer Center Institute for Cancer Control, Tokyo, Japan; ^2^Division of Supportive Care, Survivorship and Translational Research, National Cancer Center Institute for Cancer Control, Tokyo, Japan; ^3^Innovation Center for Supportive, Palliative and Psychosocial Care, National Cancer Center Hospital, Tokyo, Japan

**Keywords:** Implementation Mapping, implementation strategies, workplace, non-communicable diseases, health promotion, implementation science

## Abstract

**Introduction:**

Workplace programs to prevent non-communicable diseases (NCDs) in the workplace can help prevent the incidence of chronic diseases among employees, provide health benefits, and reduce the risk of financial loss. Nevertheless, these programs are not fully implemented, particularly in small- and medium-sized enterprises (SMEs). The purpose of this study was to develop implementation strategies for health promotion activities to prevent NCDs in Japanese SMEs using Implementation Mapping (IM) to present the process in a systematic, transparent, and replicable manner.

**Methods:**

Qualitative methods using interviews and focus group discussions with 15 SMEs and 20 public health nurses were conducted in a previous study. This study applied the Consolidated Framework for Implementation Research and IM to analyze this dataset to develop implementation strategies suitable for SMEs in Japan.

**Results:**

In task 2 of the IM, we identified performance objectives, determinants, and change objectives for each implementation stage: adoption, implementation, and maintenance; to identify the required actors and actions necessary to enhance implementation effectiveness. Twenty-two performance objectives were identified in each implementation stage. In task 3 of the IM, the planning group matched behavioral change methods (e.g., modeling and setting of graded tasks, framing, self-re-evaluation, and environmental re-evaluation) with determinants to address the performance objectives. We used a consolidated framework for implementation research to select the optimal behavioral change technique for performance objectives and determinants and designed a practical application. The planning team agreed on the inclusion of sixteen strategies from the final strategies list compiled and presented to it for consensus, for the overall implementation plan design.

**Discussion:**

This paper provides the implementation strategies for NCDs prevention for SMEs in Japan following an IM protocol. Although the identified implementation strategies might not be generalizable to all SMEs planning implementation of health promotion activities, because they were tailored to contextual factors identified in a formative research. However, identified performance objectives and implementation strategies can help direct the next steps in launching preventive programs against NCDs in SMEs.

## Introduction

Non-communicable diseases (NCDs) kill 41 million people each year, equivalent to 71% of all deaths globally (1). Tobacco use, physical inactivity, harmful use of alcohol, and an unhealthy diet increase the risk of dying from NCDs (1). In Japan, four of the top five leading causes of mortality in 2019 are NCDs (i.e., Alzheimer's disease, stroke, ischemic heart disease, and lung cancer), and NCDs account for more than 80% of all health losses measured using the disability-adjusted life years (2, 3). The World Health Organization has identified workplaces as valuable access points for providing interventions targeting NCD prevention (4). In effect, workplaces provide many adults with opportunities for health promotion. Workplace health promotion programs are effective in modifying dietary behavior (5), tobacco use (6), and physical activity (7, 8). Furthermore, workplaces have existing infrastructure to provide comprehensive health promotion and disease management programs (9). Thus, workplace health promotion activities could make a significant contribution to population level reductions in chronic disease risk (10, 11).

Companies in developed countries are increasingly providing workplace health promotion programs, but the implementation in small- and medium-sized enterprises (SMEs) is limited compared with that in larger companies. For example, in 2018, 82% of large firms and 53% of small enterprises in the United States offered a wellness program (12). Similarly, occupational health activities at SMEs in Japan are lagging in large companies (13). A recent national survey in Japan showed that although SMEs have become increasingly interested in workplace health promotion, only 20% are engaged in any type of health-promoting activities (14).

The challenges smaller workplaces face in offering workplace health promotion programs include having few vendors to serve them, low commitment to and internal capacity for program delivery (15), and limited direct or administrative costs of running programs (16). The identified barrier in Japanese SMEs also includes the beliefs held by the employer/manager that health management is one's own responsibility (17). Furthermore, as smaller workplaces often have high employee turnover rates, investing in workplace health promotion programs designed to prevent chronic diseases made little sense to employers (18).

New approaches are needed that are tailored to each context to overcome these barriers at SMEs. Implementation strategies are defined as “methods or techniques used to enhance the adoption, implementation, and sustainability of a clinical program or practice” (19). Empirical studies in clinical settings show that implementation strategies, such as audit and feedback (20), training (21), and academic detailing (22), improve the implementation of evidence-based policies and practices. A systematic review regarding implementation strategies to improve health promotion policies or practices at the workplace identified six studies and found no conclusive evidence regarding the effects of those strategies (23), which may be partly due to the limited use of theory to design implementation strategies (24). Four out of the six included studies reported using theoretical, practical, or conceptual frameworks; however, these studies were used to understand the context rather than for the development of implementation strategies (23). Since the process of identifying implementation strategies is not clearly documented, it is difficult to understand which strategies work and why they work (25). Therefore, identifying implementation strategies that address barriers to implementation after a comprehensive formative evaluation with theoretical frameworks may be the most effective approach for maximizing the impact of implementation strategies in the workplace (23).

Implementation Mapping (IM) is derived from intervention mapping, which is one of the several methods (concept mapping, group model building, conjoint analysis, intervention mapping, etc.) that can be used to select implementation strategies to address the barriers and facilitators of specific evidence-based practices (26). Specifically, IM identifies implementation strategies that have the greatest potential impact on implementation and health outcomes and addresses the barriers to implementation after a comprehensive formative evaluation using theoretical frameworks (27). Moreover, IM can provide a systematic process for selecting the implementation strategies needed to overcome the barriers to implementation (27). The use of a systematic process has the advantage of increasing reproducibility, and the use of relevant theory has the advantage of increasing the likelihood of identifying the mechanism of action of implementation strategies (25). Therefore, in this study, we decided for IM as it can be used to systematically design implementation strategies. The purpose of this study was to develop implementation strategies for health promotion activities to prevent NCDs in Japanese SMEs using IM, to present the process in a systematic, transparent, and replicable manner.

## Methods

### Theoretical framework

**Figure 1:**
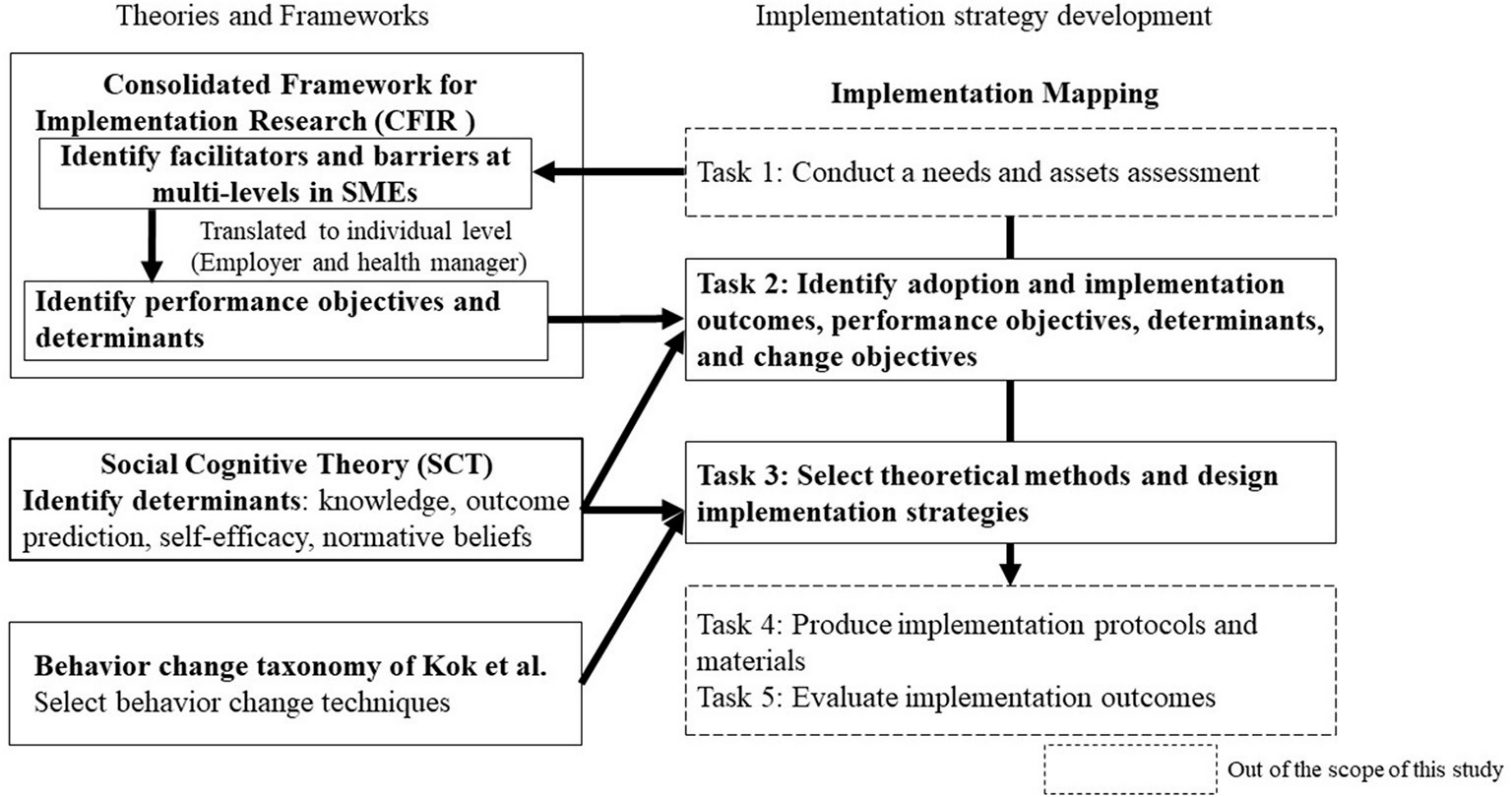
Conceptual framework.

In this study, we designed the implementation strategies for health promotion activities to prevent NCDs by using the IM framework, the Consolidated Framework for Implementation Research (CFIR) (28), social cognitive theory (29), and behavioral change taxonomy of Kok et al. (30) (Figure 1).

We selected evidence-based interventions that public health nurses as external change agents could support for implementation in the workplace in Japan: modifying dietary behavior (e.g., menu modification at cafeteria with nutrition education) (5), tobacco use (e.g., in combination with counseling, pharmacological treatment, and smoke-free polices) (6), and physical activity (e.g., physical activity program with pedometer delivery and tailored e-mail message) (7, 8).

The IM process consisted of five tasks: tasks 1 to 5. In this study, we used tasks 2 and 3 to develop implementation strategies for the adoption, implementation, and maintenance of workplace cancer prevention programs (Figure 1). CFIR, a meta-framework, includes five domains: intervention characteristics, outer setting, inner setting, characteristics of individuals, and the process (28). We used CFIR because it is important to have a comprehensive understanding of the barriers and facilitators affecting the implementation process at different levels in SMEs, which can then be used to identify context-specific implementation strategies (17). In this study, we used CFIR primarily to identify performance objectives and determinants for task 2. Similarly, we also used the social cognitive theory model (29), which can identify personal determinants and predictive relationships that promote implementation behavior, to identify the determinants of task 2. In task 3, behavioral change techniques had to be logically followed based on the determinants (27). Therefore, we used the behavior change taxonomy provided by Kok et al. (30) as prominent health behavior theories are known to influence behavioral determinants. The social cognitive theory was also used as a reference when selecting the method of behavioral change.

### Task 1: Conduct needs and assets assessments and identify actors

Task 1 was conducted prior to this study and has been published as an original publication (17). In this previous study, we identified several barriers and facilitative factors of SMEs using CFIR through the semi-structured interviews with employers and health managers (17). Semi-structured interviews were conducted with health managers and/or employers in 15 enterprises with <300 employees and four focus group discussions with 20 public health nurses/nutritionists at the Japan Health Insurance Association (JHIA) branch offices that support SMEs in four prefectures across Japan. In the previous study, we reported that of the 39 CFIR constructs, 25 were facilitative and 7 were inhibitory for workplace health promotion implementation in SMEs at individual, internal, and external levels. In particular, the leadership engagement of employers in implementing the workplace health promotion activities was identified as a fundamental factor that may influence other facilitators, including “access to knowledge and information,” “relative priority,” and “learning climate” at organizational level, as well as “self-efficacy” at the health manager level. The main barrier was the beliefs held by the employer/manager that “health management is one's own responsibility” (17). Thereafter, we identified employers and health managers as actors because health managers are the implementers of health promotion activities, and employers have the greatest influence on SMEs. Thus, we aimed to develop implementation strategies targeting employers and health managers. In this study, we translated the barriers and facilitators identified in the previous study (17) at the individual level and used them primarily to identify performance objectives and determinants for task 2.

### Formation of an implementation strategy planning team

We formed an implementation strategy planning team to guide the IM process. The group consisted of an academic team whose members specialized in psychology, public health, and epidemiology, as well as three public health nurses with at least 10 years of experience in workplace health promotion activities affiliated with the JHIA. JHIA is the largest medical insurer in Japan covering ~2.4 million enterprises (31, 32). Since most of the member companies of JHIA are SMEs (33), JHIA represents the insurers of SMEs, and more than 90% of them have <30 employees (33). In Japan, public health nurses work at various health care facilities, including publicly funded or government health insurance associations that provide health care services for workers in SMEs (34). In addition, public health nurses have recently been providing support to promote health promotion activities in SMEs and in envisioning enterprises that are members of the JHIA, as sites for implementation. We held discussions with the JHIA head office and obtained their agreement and full cooperation to promote health promotion activities in SMEs. Considering this background of public health nurses' activities in Japan along with the previous research and literature reviews conducted by the academic team and the importance of JHIA's role in scaling up the intervention, we pre-determined public health nurses affiliated with the JHIA as stakeholders for the adoption, implementation, and maintenance of health promotion activities.

### Task 2: Identifying adoption, implementation, and maintenance outcomes; performance objectives; determinants; and change objectives

In task 2, we identified the program-use outcomes and the performance objectives for each implementation stage as adoption, implementation, and maintenance because the actor who adopts, and those who implements and maintains programs will be often different. First, we determined the program-use outcomes based on each implementation stage definition (35): “adoption is the decision to use a new program; implementation is the use of the program over a long enough period to allow for evaluation regarding the innovation and whether it meets the perceived need; and maintenance is the extent to which the program is continued, and then becomes a part of normal practices.” We then selected the performance objectives necessary to achieve the program-use outcomes. The performance objectives denoted specific behaviors of those who needed to act if the change was to occur. As such, the performance objectives are action-oriented and do not include cognitive processes such as knowing and believing (27). To formulate the determinants, we used the barriers identified in task 1 and social cognitive theory (Figure 1). The academic team developed draft performance objectives that should be achieved by employers and health managers to implement the programs based on the facilitators identified in task 1, and used CFIR to provide answers to “What do the program implementers need to do to deliver the essential program components?” Since the interventionists envisioned in this workplace health promotion activities are public health nurses in JHIA, we focused on performance objectives in which public health nurses can intervene. We refined the draft performance objectives, through discussion with the public health nurses, and divided them into implementation stages of adoption, implementation, and maintenance to achieve the program-use outcomes. We then sought input from the SMEs employers and health managers who participated in the task 1 interviews, and selected performance objectives based on the feasibility, especially in terms of financial and human resources. This was done to overcome one of the barriers to implementing health promotion programs in SMEs: low available human resources and limited economic costs (15, 16). Subsequently, a matrix based on the combination of performance objectives and individual determinants of the theory of action was created. Next, we identified the personal determinants of the actors. Determinants answered the question “why,” and the barriers and facilitators of adoption were also deemed as determinants (27). We identified the determinants for each stage in a brainstorming session where the academic team answered the questions, “why do employers not understand their employees' health issues?” and “why are employers not making workplace health promotion activities a priority?” Therefore, we derived the personal determinants from the barriers identified in task 1 and the social cognitive theory model (29). In Tables 1–3, the second column of the matrix contains the performance objectives, while the other column headings are the determinants. The change objectives required to achieve each performance objective are listed under the headings in the determinant's column of the matrix. Three different matrices were created for each implementation stage of the program: Adoption (Table 1), Implementation (Table 2), and Maintenance (Table 3). In developing the matrices for task 2, the academic team held weekly discussions to reach a consensus and asked the employers and health managers of the SMEs who participated in the interviews in task 1 to share their opinions on the draft performance objectives. We sent an email to the SMEs with a draft of the performance objectives, followed by a 30-min telephonic interview with each SME. We then spent a month to make decisions after two 1-h discussions with the public health nurses. Specifically, the academic team developed a draft matrix, held online meetings with public health nurses, and revised the matrix, confirming that the change objectives were feasible and capable of achieving the performance objectives.

**Table 1:** Implementation Mapping process Task 2: Adoption.

**Program-use outcomes**	**Performance objective**		**Knowledge**		**Attitude**		**Outcome expectations**		**Self-efficacy**		**Normative beliefs**	
Adoption: Choose a suitable health promotion activity	PO1	The employer and health manager understand employees' health issues.	K1	Recognize the types and proportions of health issues faced by employees and specify risks when leaving them without addressing.	A1	Perceive the importance of understanding employees' health issues.	OE1	Expect that understanding the health issues of employees makes it smooth to introduce the health promotion program.	SE1	Demonstrate confidence in the ability to understand employee's health issues.	NB1	Believes that understanding employee health issues is a required role for employers and health managers.
	PO2	The employer agrees with the need for employees' health promotion.	K2	Defines the benefits of introducing health promotion and the risks when it is not introduced.	A2	Describes the importance of improving employees' health for the sake of the company.	OE2	Expects positive changes in employees' health and performance by health promotion.	SE2	Expresses confidence in the ability to implement health promotion.	NB2	Believes that the employers in other companies agree on health promotion.
	PO3	The employer appoints a health manager to improve employees' health as part of his/her duties.	K3	Describes the benefits when introducing health promotion activities.	A3	Recognizes that it is important for health managers to be responsible for health promotion in their work.	OE3	Expects that health promotion activities will improve employees' health.	SE3	Demonstrates the ability to get the health manager to take on health promotion as part of their work.	NB3	Believes that initiating health promotion as part of the health manager's duties is a role the employers should perform.
	PO4	The employer builds a relationship of trust with the health manager.	K4	Describes the impact of a good relationship between the employer and the health manager on project promotion.	A4	Describes that a good relationship between the employer and the health manager is important for promoting/proceeding with the project.	OE4	Expects that the good relationship between the employer and the health manager will improve the project's progress.	SE4	Demonstrates the ability to improve the relationship between the employer and the health manager.	NB4	Perceive that building a good relationship between employers and health managers is essential for introducing health promotion activities.
	PO5	The health manager builds cooperation with public health nurses.	K5	Defines the benefits of cooperation with a public health nurse during the company's health promotion initiatives.	A5	Perceives that cooperation with public health nurses is important for the health promotion of the company.	OE5	Expects that cooperation with a public health nurse will improve the health promotion of the company.	SE5	Demonstrates confidence in the ability to cooperate well with public health nurses.	NB5	Recognizes that cooperation between health managers and public health nurses is also practiced by other companies.
	PO6	The employer and health manager understand the details of intervention for the health promotion activity (e.g., physical activity, programs for reducing hypertension, and encouragement to quit smoking).	K6	Describe the intervention used in the health promotion activity in detail.	A6	Understand the importance of comprehending the details of intervention to the health promotion activity.	OE6	Expect selecting the best activity for the company by understanding interventions for health promotion activity in detail.	SE6	Demonstrate confidence in being able to understand the details of the intervention regarding the health promotion activity.	NB6	Recognize that understanding the interventions related to health promotion activities is a role of employers and health managers.
	PO7	The employer identifies the resources (human resources, costs, and goods) required to implement the health promotion activity.	K7	Defines funding flow, available resources, and required resources.	A7	Perceives that the identification of resources that will be needed and the funding flow is important to determine health promotion activity.	OE7	Expects that identifying the funding flow and available resources will facilitate the decision to implement health promotion activity.	SE7	Demonstrates the ability to identify funding flows and available resources.	NB7	Recognize that it is the role of the employers to clarify funding flow and available resources.
	PO8	The health manager selects the health promotion activity to introduce in the company.	K8	Defines which health promotion activities are appropriate to solve the health issues in the company.	A8	Perceives that choosing the suitable health promotion activity is important for solving health problems and facilitates convincing the employees for introducing the activity.	OE8	Expects that it is possible to improve employees' health if the health promotion activities chosen are appropriate.	SE8	Expresses confidence in the ability to choose the appropriate health promotion activity.	NB8	Believes that selecting the most appropriate health promotion activities is a required role of health managers.
	PO9	The employer agrees to introduce health promotion activities.	K9	Defines the impact of health promotion activities on company health promotion.	A9	Perceives that the optimal health promotion activity is important for improving employees' health and increasing company productivity.	OE9	Expects that appropriate health promotion activity will lead to improvement in employees' health.	SE9	Expresses confidence in the ability to introduce the health promotion activity.	NB9	Recognizes that selecting appropriate health promotion activities is a role expected of employers by employees.

**Table 2:** Implementation Mapping process Task 2: Implementation.

**Program-use outcomes**	**Performance objective**		**Knowledge**		**Attitude**		**Outcome expectations**		**Self-efficacy**		**Normative beliefs**	
Implement the suitable health promotion activity	PO10	The employer and the health manager receive the evidence based knowledge about the intervention of the health promotion activity to be implement.	K10	Define the benefits of gaining knowledge of the intervention.	A10	Perceive that it is important for the employer and the health manager to have the correct knowledge about the intervention in implementing the activity.	OE10	Expect that the health promotion activity can be implemented smoothly if the employer and the health manager acquire evidence-based knowledge about the intervention.	SE10	Demonstrate confidence in the ability to acquire the evidence based knowledge about interventions.	NB10	Believes that employers and health managers at other companies are also obtaining evidence-based knowledge.
	PO11	The employer facilitates employee communication.	K11	Define smooth communication between the employer and employees.	A11	Perceive that communication between the employer and the employees is important for facilitate health promotion activities.	OE11	Expect that smooth communication between the employer and employees will facilitate implementation of the suitable health promotion activity.	SE11	Demonstrate confidence in the ability to facilitate communication between the employer and employees.	NB11	Recognize that smooth communication with employees is a required behavior of employers.
	PO12	The health manager facilitates employee communication.	K12	Define the benefits of smooth communication between personnel and employees.	A12	Describe that communication between the health manager and employees is important for advancing health promotion.	OE12	Expect that smooth communication between the health manager and employees will facilitate the advancement of health promotion.	SE12	Demonstrate confidence in the ability to facilitate communication between personnel and employees.	NB12	Recognizes that smooth communication with employees is a required behavior of health managers.
	PO13	The health manager grasps the needs of employees in implementing the health promotion activity.	K13	Explain that understanding the needs of your employees will make it easier to proceed with the activity.	A13	Perceive that understanding the needs of employees is important in implementing the health promotion activity.	OE13	Expect that understanding the needs of employees in implementing health promotion activity will lead to an increase in the level of implementation.	SE13	Express confidence that gathering employees' needs for health promotion activities will be successful.	NB13	Recognizes that assessing the needs of employees is required behavior of health managers.
	PO14	The health manager customizes interventions of health promotion activity to meet employee needs.	K14	Define customization of interventions to meet the needs of employees.	A14	Perceive that it is important to customize interventions to meet the needs of employees.	OE14	Expect to increase the rate of health promotion activity implementation by providing interventions tailored to employees' needs.	SE14	Express confidence that you have the ability to customize according to needs.	NB14	Recognize that customizing to needs is a required role of a health manager by employees.
	PO15	The employers put health promotion activity as a priority.	K15	Define the benefits of putting health promotion programs as a priority.	A15	Perceive that it is important to put health promotion programs as a priority in the implementation of the health promotion activity	OE15	Expect that prioritizing health promotion programs will increase the implementation rate of health promotion and improve the health of employees.	SE15	Demonstrate confidence that put health promotion programs as a priority	NB15	Believe that other companies with successful health promotion prioritize health promotion programs
	PO16	The employer and the health manager set the purpose and goal of implementing the health promotion programs.	K16	Define the purpose of health promotion activity implementation and the benefits of setting goals.	A16	Describe that setting the purpose and goal of activity implementation in order to implement the health promotion activity is important	OE16	Expect that the implementation rate will increase upon setting the purpose and goal of activity implementation.	SE16	Demonstrate confidence in ability to set goals for activity implementation	NB16	Recognize that employer should set goals before activities are implemented.
	PO17	The employers declare to employees the purpose and goals of implementing the health promotion activity.	K17	Define the significance of the employer to declare the purpose and goals to employees.	A17	Perceive that it is important for the employer declare to the purpose and goals to employees in implementing the health promotion activity.	OE17	Expect that the implementation rate will increase and the health of employees will improve if the employer will declare the purpose and goals to employees.	SE17	Demonstrate confidence that employers can declare health promotion goals and purposes to employees.	NB17	Recognize that declaring objectives and goals to employees is a role expected of employers by employees.
	PO18	The health manager customizes the evidence based information and delivers it to the employee.	K18	Define the benefits of getting evidence-based information, and define the benefits of customizing the information.	A18	Perceive that it is important for activity implementation to customize and deliver evidence-based information.	OE18	Expect that the understanding and knowledge of employees and the activity implementation rate will increase by customizing and delivering evidence-based information.	SE18	Demonstrate the ability to customize and deliver evidence-based information.	NB18	Believes that providing customized, evidence-based information to employees is a role of health managers.
	PO19	The health manager finds a champion.	K19	Define the benefits of the existence of a champion.	A19	Perceive that the presence of a champion is important for health promotion activity implementation.	OE19	Expect to have a positive impact on employee health by finding a champion.	SE19	Express confidence that you can find a champion.	NB19	Recognize that finding champions is a role of the health managers.
	PO20	Employers create connections with other companies to exchange information on health promotion.	K20	Define the benefits of create connections with other companies to exchange information on health promotion.	A20	Believe that exchanging information with other companies is important for implementation health promotion activity.	OE20	Expect to be able to implement good practices in their own companies by exchanging information with other companies.	SE20	Express confidence that you can exchange information with other companies.	NB20	Recognize that other employers with successful health promotions are also exchanging information with other companies.

**Table 3:** Implementation Mapping process Task 2: Maintenance.

**Program-use outcomes**	**Performance objective**		**Knowledge**		**Attitude**		**Outcome expectations**		**Self-efficacy**		**Normative beliefs**	
Sustain the suitable health promotion activity	PO21	The health manager sets medium- to long-term goals.	K21	Define the benefits of setting medium- and long-term goals.	A21	Perceive that setting medium- to long-term goals is important for continuing health promotion activity.	OE21	Expect that the sustainability of health promotion activity implementation will increase by setting medium- to long-term goals.	SE21	Demonstrate confidence in the ability to set medium- to long-term goals.	NB21	Recognize that it is the role of the health managers to set mid- to long-term goals.
	PO22	The health manager creates an evaluation mechanism and rotates the PDSA cycle.	K22	Create an evaluation mechanism and define the benefits of running the PDSA cycle.	A22	Understand that it is important to maintain the health promotion activity by creating an evaluation mechanism and rotating the PDSA cycle.	OE22	Expect to maintain a health promotion activity by building an evaluation system and implementing a PDSA cycle.	SE22	Express confidence to create an evaluation system and rotate the PDSA cycle.	NB22	Recognize that it is essential for health managers to create a system of evaluation and to run the PDSA cycle to maintain health promotion activities.

Abbreviations: PDSA, Plan-Do-Study-Act.

### Task 3: Select theoretical methods and design implementation strategies

Task 3 aimed to select a theoretical method and design implementation strategies. We selected suitable behavioral change techniques using the behavior change taxonomy of Kok et al. (30) for each determinant of the matrix created in task 2. This taxonomy outlines ways to change perceptions, attitudes, beliefs, outcome expectations, skills, abilities, self-efficacy, environmental conditions, social norms, social support, organizations, communities, and policies. In selecting behavioral change techniques, as in task 2, the academic team created a draft and revised it through online or in-person discussions with the public health nurses. These discussions were held over the course of a month and involved two 1-h discussions with the public health nurses on two occasions.

## Results

The results are presented by IM task.

### Task 2: Identify adoption, implementation, and maintenance outcomes; performance objectives; determinants; and change objectives

For this task, we identified the program-use outcomes, performance objectives (“What had to be done by whom to implement the program?”), determinants (“Why would an actor perform the program as planned?”), and change objectives (“What has to change in this determinant in order to bring about the performance objective?”), for each implementation stage. Tables 1–3 show the program-use outcome, the subsequent specific steps required to meet them (i.e., performance objectives), determinants, and change objectives for each implementation stage. For the adoption stage, we set the program-use outcome as “choosing health promotion activities that are suitable for the company's health issues.” Therefore, we set the performance objectives as the process of team building to adopt health promotion activities, such as “employer identification of employee's health issues” and “building trust between employers and health managers.” We selected these performance objectives from the facilitators at the “inner setting” and “process” CFIR domains (in particular “readiness for implementation,” “implementation climate,” and “formally appointed internal implementation leaders”) (Table 1).

We set the program-use outcome for the implementation stage as implementing health promotion activities appropriate to the company's health issues (Table 2). For instance, we chose the performance objective to include the health manager assessing the needs of the employees and customizing the intervention, and the employer setting the objectives and goals of the health promotion activities, and declaring them to the employees. We selected these from the “outer setting” (e.g., “needs and resources of those served by the organization”) and “inner setting” (especially “leadership engagement” and “goals and feedback”) facilitators of the CFIR domains. In addition, we also chose “employers to connect with other businesses and exchange information on health promotion” for the performance objectives, based on information from the CFIR domain “cosmopolitanism.” We set the program-use outcome for the maintenance stage to sustain health promotion activities (Table 3). Therefore, we chose the performance objectives to include mid-to long-term goal setting and evaluation of health promotion activities. These were selected from the facilitators of the “process” (“reflecting and evaluating”) CFIR domain.

Subsequently, we identified the determinants of the barriers to task 1 and social cognitive theory. The primary barrier was the belief held by the employers or managers that “health care is a self-responsibility” with information from the CFIR domain characteristics of individuals (17). We adopted this as a determinant factor as “attitude”, which implies a low awareness of the importance of health promotion activities in the workplace. Furthermore, from the theoretical determinants of the social cognitive theory, we employed knowledge, outcome prediction, self-efficacy, and normative beliefs as the determinants of relevance for performance objective.

With the performance objectives and determinants established, task 2 outcomes were used in the creation of the matrix of change objectives for each stage. We identified 22 performance objectives and 5 determinants (i.e., knowledge, attitudes, outcome expectations, self-efficacy, and normative beliefs). Change objectives (written where the matrix rows and columns intersect) reflected the changes in the five determinants that were needed for the performance objectives to be completed successfully for each implementation stage of health promotion activities. We received opinions from the employers and health care managers, primarily for performance objectives, whether they were appropriate to achieve program use outcomes in each implementation stage, and whether they were feasible with the support of public health nurses. The public health nurses advised the academic team, based on their experience in health promotion support activities, to set feasible performance objectives with respect to cost and human resources. The academic team revised and finalized the performance objective based on their advice.

### Task 3: Select theoretical methods and design implementation strategies

The planning team selected discrete implementation strategies to operationalize performance objectives.

First, we selected behavioral change techniques from the taxonomy of behavioral change methods (30) (e.g., modeling and setting of graded tasks [social cognitive theory], framing [protection motivation theory], self-re-evaluation, and environmental re-evaluation [transtheoretical model]). These behavioral change techniques were selected according to the following three criteria: (1) the interventionists could use convincing language to encourage the adoption and implementation of the program, (2) the methods could be used even by non-expert health professionals, and (3) they considered the real-life work environment and Japanese culture. We decided on these criteria through discussions with the public health nurses.

Second, we selected behavioral change techniques for each determinant regarding social cognitive theory and designed practical applications. For example, the behavioral change technique, modeling, is known to be associated with normative beliefs, outcome expectations, and self-efficacy (29).

Information on health promotion activities in other SMEs could improve organization leadership's receptiveness to adopting workplace programs. Furthermore, information on the role of other employers in health promotion activities could help them acquire their own role models and predict positive outcomes. Therefore, modeling was selected as a method of behavioral change for the determinants of normative beliefs, outcome expectations, and self-efficacy. We then designed the practical application of modeling to address the performance objective-14 as, “to provide employers with precedents of how their own health promotion activities have been successful as a result of sharing information regarding health promotion activities with other companies.” In addition, the interventionist would explain that it is desirable for employers to take the lead in creating relationships with other companies (Table 4). This task was completed in 1 month with the planning team meeting weekly to review the outputs of task 3, review and discuss the literature, and iteratively update the list of change methods and practical applications. The team discussed the determinants most strongly associated with each performance objective and agreed to include 16 discrete strategies in the overall implementation plan design. Table 4 summarizes the agents, determinants, methods of change, and discrete strategies used according to the implementation phase of the health promotion activities in the implementation strategies. In addition, to compare with previous reviews, the academic team discussed and reached a consensus on where the practical application corresponds to the Expert Recommendations for Implementing Change (ERIC) taxonomy and included it in Table 4.

**Table 4:** Implementation strategies in health promotion activities within small- to medium-enterprises.

**Stage**	**No**.	**Actor**	**Performance objective**	**Determinants and change objectives**	**Theoretical method (parameters)**	**Practical application**	**ERIC**
Adoption	1	Employer/health manager	PO1. Understand employee health issues.	Knowledge: Recognize the types and proportions of health issues faced by employees and define the risks of leaving them unattended.	Framing (Requires high self-efficacy expectations.)	Intervenors emphasize the many benefits and effectiveness of employers' understanding of employees' health issues in conducting health promotion activities.	Use evaluative and iterative strategies
	2	Employer	PO2. Agrees with the need for employee health promotion.	Attitude: Recognize the importance of improving employee's health for the sake of the company.	Environmental re-evaluation (May include awareness about serving as a role model for others.)	Discuss with public health nurses and health manager and recognize the wide range of impacts of whether or not to engage in health promotion activities in the workplace.	Develop stakeholder interrelationships
	3	Employer/health manager	PO4. Builds a relationship of trust with the health manager.	Normative beliefs: Perceives that building a good relationship between employers and health managers is essential for the introduction of health promotion activities.	Belief selection (Requires investigation of the current attitudinal, normative and efficacy beliefs of the individual before choosing the beliefs on which to intervene.)	Interveners explain that when implementing workplace health promotion activities, it is important for employers and health managers to share the same beliefs and collaborate.	Develop stakeholder interrelationships
	4	Health manager	PO5. Builds cooperation with public health nurses.	Attitude: Perceives that cooperation with public health nurses is important for the health promotion of the company.	Forming coalitions (Requires collaboration across various agendas; requires attention to stages of partnership development.)	Interveners will make the health manager aware that building a partnership with the public health nurse can make a difference in the rate of implementation of health promotion, and will mediate the relationship building.	Develop stakeholder interrelationships
	5	Employer	PO9. Agree with the need for employee health promotion.	Outcome Expectations: Expect positive changes in employees and business performance by promoting health.	Self-re-evaluation (Stimulation of both cognitive and affective appraisal of self-image.)	Interveners will explain the significant role that employers play in health promotion activities and the positive impact on the company.	Develop stakeholder interrelationships
Implementation	6	Employer/health manager	PO10. Get the evidence-based knowledge regarding the intervention of the health promotion activity to be implement.	Attitude: Perceive that it is important for the employer and the health manager to have the correct knowledge regarding the intervention in implementing the activity.	Environmental re-evaluation (May include awareness about serving as a role model for others.)	Interveners will explain the impact of actors obtaining or not obtaining appropriate evidence-based knowledge and encourage knowledge acquisition.	Train and educate stakeholders
	7	Employer	PO11. Facilitate communication with employees.	Self-efficacy: Show confidence that employee communication can be facilitated.	Modeling (Appropriate models will vary by target.)	Interveners will facilitate communication between the employer and the health manager by using precedents of similarly sized companies and other companies in the same industry to facilitate discussion.	Engage consumers
	8	Health manager	PO13. Understand the needs of employees in implementing the activity.	Self-efficacy: Be confident that you can successfully assess employees' needs in implementing the activity.	Set graded tasks (The final behavior can be reduced to easier but increasingly difficult sub-behaviors.)	Interveners facilitates the health manager to list and take actions necessary to identify needs for health promotion of employees.	Use evaluative and iterative strategies
	9	Health manager	PO14. Customize interventions to meet employees;' needs.	Normative beliefs: Recognize that customizing to needs is a required role of a health manager by employees.	Environmental re-evaluation (May include awareness about serving as a role model for others.)	Interveners will ask the health manager how the employee perceives and feels regarding the health manager who will/will not customize (intervene) to the employee's needs. Then, through discussion with the health manager, make the health manager aware that customizing health promotion activities to their needs is the ideal behavior.	Adapt and tailor to context
	10	Employer	PO15. Make health promotion activity as a priority.	Self-efficacy: Demonstrate confidence that put health promotion activities as a priority	Reinforcement (Reinforcement need to be tailored to the individual, group, or organization.)	Interveners will identify measures that employers have prioritized to improve health and benefit employees, highlighting their experiences and providing positive feedback.	Change infrastructure
	11	Employer/health manager	PO16. Set the purpose and goals for health activity implementation.	Outcome Expectations: Expect that the implementation rate will increase by setting the purpose and goal of activity implementation.	Modeling (Appropriate models will vary by target.)	Interveners will provide information on precedents where health promotion activities have been successfully developed with appropriate goal setting and will facilitate goal setting.	Use evaluative and iterative strategies
	12	Employer	PO17. Declare the purpose and goals of the health activity to employees.	Self-efficacy: The employer is confident that he can directly convey the purpose and goals of health promotion to the employees and resonate with them.	Set graded tasks (The final behavior can be reduced to easier but increasingly difficult sub-behaviors.) /Provide contingent rewards (Rewards need to be tailored to the target.)	Interveners will identify graded tasks, such as preparing manuscripts and conducting role-plays and enable employers to successfully implement the health declaration. Positive feedback is given when tasks are successfully completed.	Change infrastructure
	13	Health manager	PO18. Customize evidence-based information and deliver it to employees.	Normative beliefs: Believes that providing customized, evidence-based information to employees is a role of health managers.	Information about others' approval (Positive expectations are available in the environment.)	Interveners instructs the health manager to devise a method of providing the information (e.g., make the letters larger in the areas to be emphasized, mark them in a prominent color, write the subject's name on them and distribute them, etc.). Then, provide feedback on the comments received from employers and employees.	Engage consumers
	14	Employer/health manager	PO20. Create connections with other companies to exchange information on health promotion.	Normative belief: Recognize that other employers with successful health promotion are also exchanging information with other companies.	Modeling (Appropriate models will vary by target.)	Interveners will provide employers with precedents of how their own health promotion activities have been successful as a result of sharing information about health promotion activities with other companies. The interventionist will explain that it is desirable for employers to lead the way in creating relationships with other companies.	Develop stakeholder interrelationships
Maintenance	15	Health manager	PO21. Set medium- to long-term goals.	Normative beliefs: Recognize that it is the role of the health managers is to set mid- to long-term goals.	Cultural similarity (Using surface characteristics of the target group enhances receptivity.)	Interveners explains that setting medium- and long-term goals is an action that should be taken as a health manager, based on prior examples of companies that are similar in size, structure, and philosophy and that do not compete with the target establishments.	Use evaluative and iterative strategies
	16	Health manager	PO22. Create a mechanism for evaluating measures and running the PDSA cycle.	Outcome Expectations: Expect to maintain a better activity by creating an evaluation mechanism and rotating the PDSA cycle.	Shifting perspective (Initiation from the perspective of the learner; needs imaginary competence.)	Interveners asks the health manager to consider a shift in perspective, specifically discussing what you would do to structure an evaluation if you were an employer or another employee or what you would advise if you were consulted by a colleague about circulating a PDSA.	Use evaluative and iterative strategies

Abbreviations: ERIC, Expert Recommendations for Implementing Change; PDSA, Plan-Do-Study-Act.

## Discussion

In this paper, we described how we developed implementation strategies for health promotion activities to prevent NCDs in SMEs. Sixteen strategies for implementing health promotion activities were developed from multiple perspectives of employers and health managers from SMEs, public health nurses, and researchers, including how to improve the programs, while receiving feedbacks from within and outside the company and being aware of social desirability.

In this study, we selected discrete implementation strategies according to the context and determinants of the organizations. Implementation strategies have different effects depending on the determinants (barriers and facilitators) (36), and the context and barriers to implementation need to be properly understood to select strategies that best address them (37). Moreover, we involved the stakeholders, the headquarters of JHIA, to build the strong partnerships needed for implementation. Strong partnerships must be necessary when it comes to changing organizational-level systems (38). For example, when considering methods to change physician behaviors, individual doctors cannot be expected to change without corresponding changes in healthcare teams and the overall organization (39). Likewise, in this study, partnership with public health nurses in JHIA was an essential element because the implementation of health promotion activities requires system changes that need to be integrated into the usual workflows at the organizational level, and also the importance of JHIA's role in scaling up the intervention in the future.

Moreover, the discrete implementation strategies we derived through IM have been reported in a systematic review of implementation strategies (23) as follows: the “develop stakeholder interrelationships” (40) (e.g., the employer agrees with the need for employees' health promotion and the health manager builds cooperation with public health nurses) in the adoption phase of our intervention; “train and educate stakeholders” (40) (e.g., the employer and health manager receive the evidence-based knowledge about the intervention of the health promotion activity to be implemented) in the implementation stage; and use evaluative and iterative strategies” (40) (e.g., the health manager sets medium- to long-term goals) in the maintenance stage. These consistencies with well-established barriers and strategies enhance the validity of our process and results and predict a degree of generalizability to other settings.

However, we identified two implementation strategies that were not found in the previous systematic review. The first strategy was to “engage consumers” (40), which is related to attentiveness and communication. For example, the health manager at SMEs customizes the content and delivery methods of evidence-based information according to the characteristics of each employee. This strategy would reflect the advantage of SMEs, which is more accommodating (16) and provides a more intimate work culture due to fewer employees, thus encouraging employees to participate in health promotion activities (41).

The second strategy involves “change in infrastructure” (40), wherein employers prioritize health promotion programs and establish the purpose and goals of implementing health promotion activities among their employees. Furthermore, it involves the “development of stakeholder interrelationships” (40), wherein employers build connections with other companies to exchange information on health promotion in the workplace; and this may generate a modeling effect across companies. These strategies, newly identified in our study, appear to reflect the Japanese culture. The declarations made by employers have a strong impact on Japanese employees, who tend to be obedient to their superiors. In the interviews conducted as part of our previous study, there was an opinion that the progress of the business would be different if there was “a word from the top” or the employer (17). In addition, the creation of horizontal connections makes “modeling” possible and makes it easier to create behavioral changes with an awareness of social norms. In Asian societies, especially in Japan, social norms are strict, with duties and obligations taking precedence (42, 43). Therefore, learning about health promotion activities in other companies generates a belief that the activities being performed in other companies should also be performed in their companies. Moreover, those norms and beliefs are often created by the opinions and attitudes of employers in SMEs. Therefore, it is an effective implementation strategy aimed at fostering the norms about health promotion activities in the company by encouraging employers to change their knowledge, attitudes, and norms.

These newly identified implementation strategies for workplace health promotion could be attributed to the focus on SMEs and the fact that we used IM to derive strategies based on real-world opinions. The implementation strategies of large businesses cannot be generalized to SMEs due to their different contexts (16), and there is a need for strategies that are optimal for the challenges faced by SMEs. Further studies to identify implementation strategies that consider the characteristics of SMEs would promote the efforts of the SMEs to overcome the barriers to the adoption and implementation of workplace health promotion.

The implementation strategies designed in this study are primarily for health promotion activities in SMEs, focusing on five NCD prevention measures (i.e., tobacco use, alcohol consumption, diet, physical activity, and health check-ups). We are currently developing protocols and materials according to task 4 of IM, which is being evaluated in a researcher-led pilot study, to implement an intervention focused on one (smoking cessation) of these five topics (44). The main focus of the workplace smoking cessation strategy is to encourage healthcare managers to encourage smokers in the workplace to quit smoking, so that SMEs with limited resources can implement it. The goal is to reduce the prevalence of smoking while providing implementation strategies tailored to the disincentive. If the pilot study confirms the effectiveness of the implementation strategies, public health nurses at JHIA will participate in the national scaling up of the program. Among employees in SMEs, the proportions of health and behavioral problems, such as hypertension, obesity, and smoking, were higher than those in employees from larger organizations (45). Therefore, employers in SMEs must make a serious effort to promote the health of their employees and prioritize health-promoting programs.

This study has several limitations. In the selection of behavioral change techniques and development of practical applications (task 3), there was insufficient involvement of SMEs. Furthermore, in task 2, employers and health managers of the SMEs were involved, but not their employees. In addition, planning with public health nurses was not a participatory approach, but rather a form of listening to their opinions. This is because it is not yet common in Japan for stakeholders in the field to be actively involved in research. Since this was our first implementation study with SMEs and JHIA, we had to be careful not to place a burden on SMEs and JHIA during this period. As a result of this background, it is possible that the opinions of the SMEs and public health nurses were not fully reflected in the field, or that they were insufficient to foster a proactive attitude among SMEs and public health nurses toward health promotion activities in the workplace. Additionally, it may take time for SMEs and public health nurses to incorporate these strategies into their workflow. This is because researcher-led implementation creates a perception of “somebody else's business,” i.e., that an external change agent, the researcher, will take care of the company's health activities.

The selection of the implementation strategies was tailored to the context of SMEs in Japan, where health promotion activities are already being implemented, and may not be effective in other settings because the strategy may not resonate with other settings, such as the limited readiness of the employer to implement the health promotion. However, in countries and communities like Japan, where the social norms influence behavior, it may be effective, but this needs to be verified.

This study developed implementation strategies for health promotion activities in SMEs in Japan by applying IM in conjunction with the constructs of the CFIR framework, social cognitive theory, and behavioral change techniques. To our knowledge, there are only a few studies that applied and integrated these three frameworks and techniques simultaneously to develop implementation strategies. The IM protocol provided a valuable guideline for the development of comprehensive implementation strategies. The identified performance objectives and implementation strategies can help direct further steps in launching health promotion activities to prevent NCDs in SMEs.

## Data availability statement

The original contributions presented in the study are included in the article/Supplementary material, further inquiries can be directed to the corresponding author/s.

## Ethics statement

The studies involving human participants were reviewed and approved by Ethical Committee of the National Cancer Center. Written informed consent for participation was not required for this study in accordance with the national legislation and the institutional requirements.

## Author contributions

MO and TS conceived of the paper and designed the study. MO, JS, AY-S, and TS were members of the academic team of the implementation strategy planning group and developed the implementation strategy according to the IM protocol. MO drafted the initial manuscript and all authors revised the manuscript for important intellectual content. TS was the principal investigator of the study. All authors read and approved the final manuscript.

## Funding

This work was supported by the National Center Consortium in Implementation Science for Health Equity (N-EQUITY) and funded by the Japan Health Research Promotion Bureau (JH) Research Fund (2019-(1)-4) and JH Project fund (JHP2022-J-02), the National Cancer Center Research and Development Fund (30-A-18 and 2021-A-19), and the Japan Society for the Promotion of Science (JSPS) KAKENHI Grant-in-Aid for Scientific Research (Grant Numbers JP21K17319 and JP22H03326).

## Conflict of interest

The authors declare that the research was conducted in the absence of any commercial or financial relationships that could be construed as a potential conflict of interest.

## Publisher's note

All claims expressed in this article are solely those of the authors and do not necessarily represent those of their affiliated organizations, or those of the publisher, the editors and the reviewers. Any product that may be evaluated in this article, or claim that may be made by its manufacturer, is not guaranteed or endorsed by the publisher.

## Abbreviations

CFIR: consolidated framework for implementation research
IM: Implementation Mapping
NCD: non-communicable disease
SMEs: small- and medium-sized enterprises.
